# Supplementary material for: Evaluation of a policy intervention to promote the health and wellbeing of workers in small and medium sized enterprises – a cluster randomised controlled trial
Source: BMC Public Health. 2019 May 2;19:493. doi: 10.1186/s12889-019-6582-y (PMC6498586; doi:10.1186/s12889-019-6582-y)
Supplement: Supplementary file 1 — Questionnaire for quantitative outcomes (employer and employee each administered separately). (DOCX 16 kb) [file 12889_2019_6582_MOESM1_ESM.docx]

**Additional file 1**

Employee questions

1. How satisfied are you with your life nowadays?

2. To what extent do you feel the things you do in your life are worthwhile?

3. How happy did you feel yesterday?

4. How anxious did you feel yesterday?

Randomised question order, 0-10 scale of not at all to completely

5. And overall, how would you rate your health?

Excellent, very good, good, fair, or poor.

6. Does your organisation take positive action on health and wellbeing? Yes / No

If YES: How much action does it take? Very little, some, quite a bit, or a lot of action?

7. In the last 6 months, has your organisation provided information about any of the following? Please select all that apply and then click next.

- Mental health?
- Musculoskeletal (MSK) health?
- Healthy lifestyles (e.g. smoking, alcohol use, diet, physical activity, active travel)?

o IF YES: Which ones of the following? Smoking, alcohol and substance misuse, healthy food and drinks, weight management, physical activity, active travel (if asked, e.g. cycling or walking to work)

Any additional comments (text): …………………………………………………………………………………………….

8. In the last six months, has your organisation provided activities or services for any of the following?

- Mental health?
- Musculoskeletal (MSK) health?
- Healthy lifestyles (e.g. smoking, alcohol use, diet, physical activity, active travel)? IF YES: Which ones of the following? Smoking, alcohol and substance misuse, healthy food and drinks, weight management, physical activity, active travel (e.g. cycling or walking to work

Any additional comments (text): …………………………………………………………………………………………….

9. In the last six months, have you made a conscious effort to improve your…

- Mental health?
- Musculoskeletal (MSK) health? (Musculoskeletal disorders can affect muscles, joints and tendons in all parts of the body. Work activities which are frequent and repetitive, or activities with awkward postures can cause or exacerbate these disorders which may be painful during work and limit work capability)
- Lifestyle health (e.g. smoking, alcohol use, diet, physical activity, active travel)?
- IF YES: Which ones of the following? Smoking, alcohol and substance misuse, healthy food and drinks, weight management, physical activity, active travel (e.g. cycling or walking to work)

Any additional comments (text): …………………………………………………………………………………………….

10a*. For those who selected ‘mental health’ in 7 or 9 above:* In previous questions, you said that your organisation provided information, activities, and/or services related to mental health. Did you use or take part in the initiative(s)? Yes/no

10b*. For those who selected ‘MSK’ in 7 or 9 above:* In previous questions, you said that your organisation provided information, activities, and/or services related to musculoskeletal (MSK) health. Did you use or take part in the initiative(s)? Yes/no

10c. *For those who selected ‘healthy lifestyles’ in 7 or 9 above:*  In previous questions, you said that your organisation provided information, activities, and/or services related to healthy lifestyles. Did you use or take part in the initiative(s)? Yes / no

- IF YES: Which ones of the following? Smoking, alcohol and substance misuse, healthy food and drinks, weight management, physical activity, active travel (e.g. cycling or walking to work)

10d. *For those who did not select mental health / MSK / healthy lifestyles in 7 or 9 above, as appropriate.* If the following initiatives were offered at work, how likely would you be to take part or use them – extremely unlikely, fairly unlikely, neutral, fairly likely or extremely likely?

- Mental health? [*This question is for the participant to answer themselves.*]
- Musculoskeletal (MSK) health? [Musculoskeletal disorders can affect muscles, joints and tendons in all parts of the body. Work activities which are frequent and repetitive, or activities with awkward postures can cause or exacerbate these disorders which may be painful during work and limit work capability]
- Healthy lifestyles (e.g. smoking, alcohol use, diet, physical activity, active travel)?
  - IF YES: Which ones of the following? Smoking, alcohol and substance misuse, healthy food and drinks, weight management, physical activity, active travel (e.g. cycling or walking to work)

Any additional comments (text): …………………………………………………………………………………………….

11. *For those who did select mental health, MSK, healthy lifestyles in 7 & 9 above as appropriate.* If the following initiatives were offered at work, how likely would you be to take part or use them – extremely unlikely, fairly unlikely, neutral, fairly likely or extremely likely?

- Mental health?
- Musculoskeletal (MSK) health?
- Healthy lifestyles (e.g. smoking, alcohol use, diet, physical activity, active travel)?

o IF YES: Which ones of the following? Smoking, alcohol and substance misuse, healthy food and drinks, weight management, physical activity, active travel (e.g. cycling or walking to work)

Any additional comments (text): …………………………………………………………………………………………….

*This question is for the participant to answer themselves.*

12. In the last six months, have you joined a group organisation or used a service that is outside your workplace related to your…

- Mental health?
- Musculoskeletal (MSK) health? (musculoskeletal disorders can affect muscles, joints and tendons in all parts of the body. Work activities which are frequent and repetitive, or activities with awkward postures can cause or exacerbate these disorders which may be painful during work and limit work capability)
- Healthy lifestyles (e.g. smoking, alcohol use, diet, physical activity, active travel)? IF YES: Which ones of the following? Smoking, alcohol and substance misuse, healthy food and drinks, weight management, physical activity, active travel (e.g. cycling or walking to work)

Any additional comments (text): …………………………………………………………………………………………….

Employer questions

1. Does your organisation take positive action on health and wellbeing? Yes / No

- If YES: How much action does it take? Very little, some, quite a bit, or a lot of action?

To what extent do you agree or disagree with the following statements?

2. The organisation is equipped to deal with staff health and wellbeing concerns

Strongly disagree --------------------------------------------Strongly agree

1 2 3 4 5

3. The organisation has an understanding of the main issues that impact on the employees’ health and wellbeing.

Strongly disagree --------------------------------------------Strongly agree

1 2 3 4 5

4. Does your organisation have updated policies/regulations (in the last 12 months) in place for any of the following? Please select all that apply.

- Bullying and harassment
- Sickness and absence
- Flexible working
- Caring responsibilities
- Whistleblowing
- Disciplinary and grievance
- Equal opportunities
- No, none of the above

Any additional comments (text): …………………………………………………………………………………………….

5. In the last 6 months, has your organisation has provided information about any of the following? Please select all that apply.

- Mental health
- Musculoskeletal (MSK) health (Musculoskeletal disorders can affect muscles, joints and tendons in all parts of the body. Work activities which are frequent and repetitive, or activities with awkward postures can cause or exacerbate these disorders which may be painful during work and limit work capability)
- Healthy lifestyles (e.g. smoking, alcohol use, diet, physical activity, active travel). IF YES: Which ones of the following? Smoking, alcohol and substance misuse, healthy food and drinks, weight management, physical activity, active travel (clarify - e.g. cycling or walking to work)

Any additional comments (text): …………………………………………………………………………………………….

6. Does your organisation provides activities or services to employees for any of the following? Please select all that apply.

- Mental health?
- Musculoskeletal (MSK) health? (musculoskeletal disorders can affect muscles, joints and tendons in all parts of the body. Work activities which are frequent and repetitive, or activities with awkward postures can cause or exacerbate these disorders which may be painful during work and limit work capability)
- Healthy lifestyles (e.g. smoking, alcohol use, diet, physical activity, active travel)? IF YES: Which ones of the following? Smoking, alcohol and substance misuse, healthy food and drinks, weight
- management, physical activity, active travel (clarify - e.g. cycling or walking to work)

Any additional comments (text): …………………………………………………………………………………………….

7a. *For those who answered ‘mental health’ to 5 or 6.* In previous questions, you said that the organisation provided information, activities and/or services related to mental health. Did your employees use or take part in the initiative(s)? Yes / no– IF YES: what proportion of your employees used or took part in the mental health initiative(s)?

7b. *For those who answered ‘MSK’ to 5 or 6.* In previous questions, you said that the organisation provided information, activities, and/or services related to musculoskeletal (MSK) health. Did your employees use or take part in the initiative(s)?

Yes / no – IF YES: what proportion of your employees used or took part in the MSK initiative(s)?

7c. *For those who answered ‘healthy lifestyles’ to 5 or 6.* In previous questions, you said that your workplace provided information, activities, and/or services related to healthy lifestyles. Did your employees use or take part in this initiative(s)?

Yes / no – IF YES: what proportion of your employees used or took part in the healthy lifestyles initiative(s)?

7d. *If yes for healthy lifestyles in 7c.* Which ones of the following for healthy lifestyles did your employees use or take part in?  Please select all that apply: Smoking, alcohol and substance misuse, healthy food and drinks, weight management, physical activity, active travel

8. *If no to mental health, MSK or healthy lifestyles in 5 or 6 as appropriate.* If the following initiatives were offered at work, how likely would your employees be to take part in or use it – extremely unlikely, fairly unlikely, neutral, fairly likely or extremely likely?

[an initiative could be providing information and/or activities or services related to mental health]

- Mental health?
- Musculoskeletal (MSK) health? [MSK - musculoskeletal disorders can affect muscles, joints and tendons in all parts of the body. Work activities which are frequent and repetitive, or activities with awkward postures can cause or exacerbate these disorders which may be painful during work and limit work capability]
- Healthy lifestyles (e.g. smoking, alcohol use, diet, physical activity, active travel)?

o IF YES: Which ones of the following? Smoking, alcohol and substance misuse, healthy food and drinks, weight management, physical activity, active travel

Any additional comments (text): …………………………………………………………………………………………….
