# Supplementary figures and images for: Evaluation of a policy intervention to promote the health and wellbeing of workers in small and medium sized enterprises – a cluster randomised controlled trial
Source: BMC Public Health. 2019 May 2;19:493. doi: 10.1186/s12889-019-6582-y (PMC6498586; doi:10.1186/s12889-019-6582-y)

**Additional file 2**

| 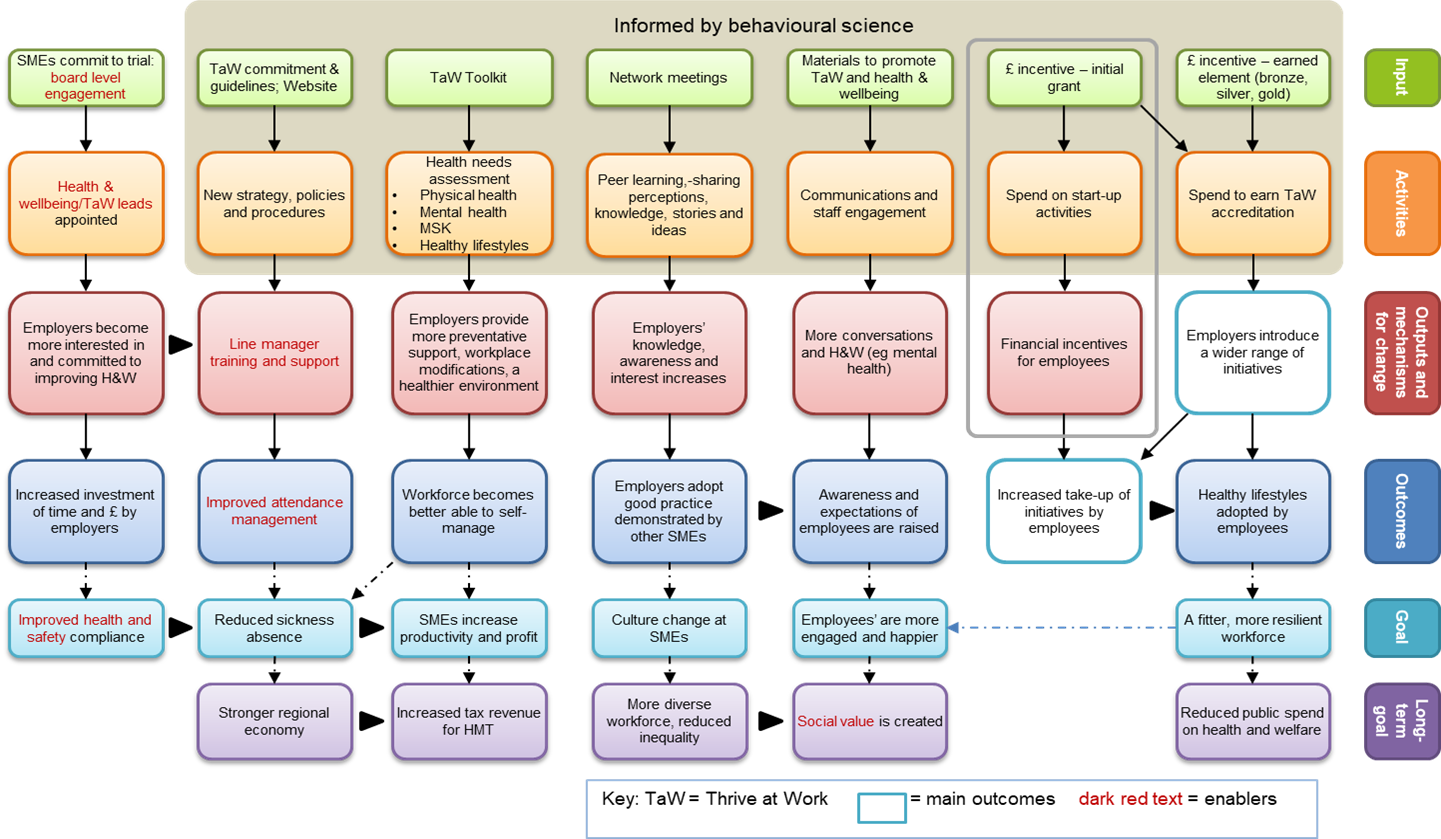 |
| --- |
| **Logic model of the Wellbeing Premium Programme or Thrive at Work** |

Supplement: Supplementary file 2 — Logic model of the Wellbeing Premium Programme or Thrive at Work used for process evaluation to capture the essential elements of the intervention and mechanisms through which the intervention is meant to work. (DOCX 606 kb) [file 12889_2019_6582_MOESM2_ESM.docx]
