# Supplementary material for: Evaluation of a policy intervention to promote the health and wellbeing of workers in small and medium sized enterprises – a cluster randomised controlled trial
Source: BMC Public Health. 2019 May 2;19:493. doi: 10.1186/s12889-019-6582-y (PMC6498586; doi:10.1186/s12889-019-6582-y)
Supplement: Supplementary file 3 — Logic model of the Wellbeing Premium Programme or Thrive at Work used for process evaluation that focuses on the intervention in the form of the financial incentives. (DOCX 84 kb) [file 12889_2019_6582_MOESM3_ESM.docx]

**Additional file 3**

| 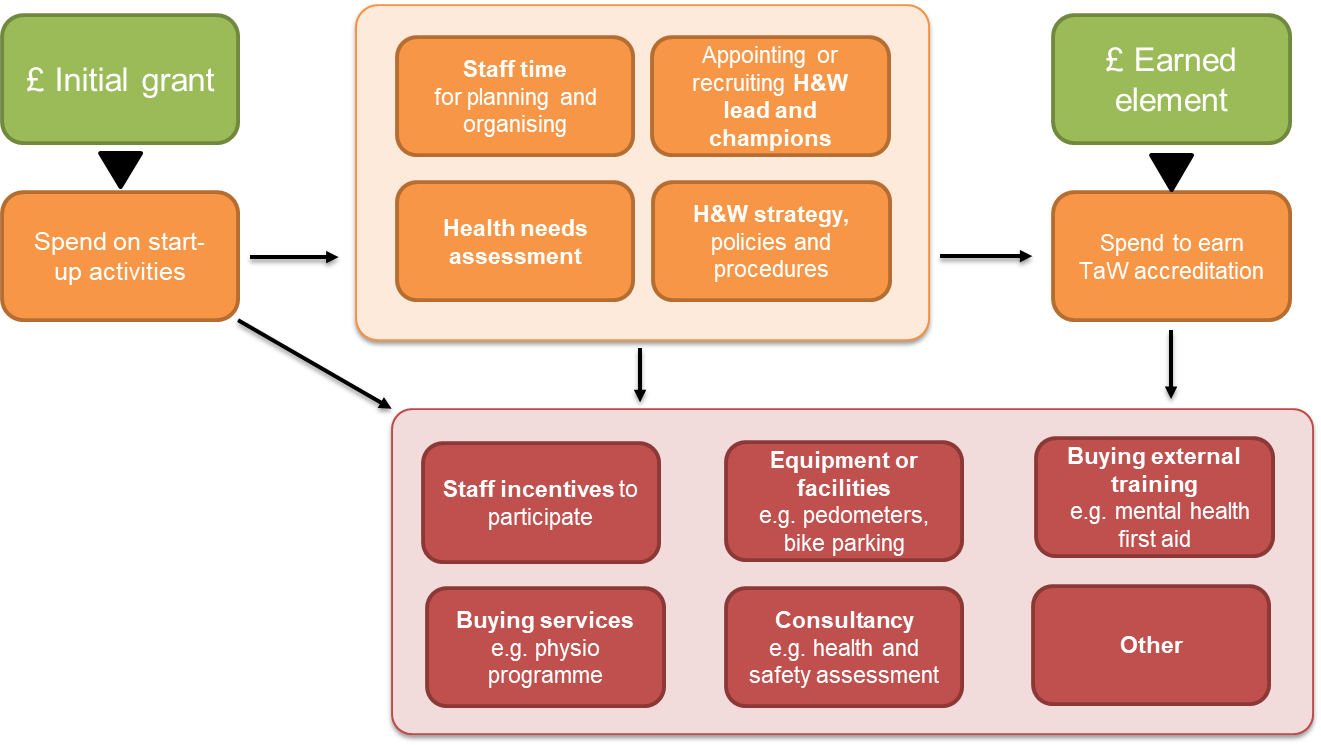 |
| --- |
| **Logic model of the Wellbeing Premium Programme or Thrive at Work – financial incentives excerpt** |
