# Supplementary material for: Evaluation of a policy intervention to promote the health and wellbeing of workers in small and medium sized enterprises – a cluster randomised controlled trial
Source: BMC Public Health. 2019 May 2;19:493. doi: 10.1186/s12889-019-6582-y (PMC6498586; doi:10.1186/s12889-019-6582-y)
Supplement: Supplementary file 4 — Description of the constrained randomisation balance metric. (DOCX 12 kb) [file 12889_2019_6582_MOESM4_ESM.docx]

**Additional file 4**

**Constrained randomisation balance metric**

The balance metric we use is Wilks’ Λ.

For each trial arm $g=1,2,3,4$ with $n_{g}$ SMEs the matrix of between group sums of squares is:

$$\boldsymbol{B}=\sum_{g=1}^{4} n_{g}\left( {\bar{\boldsymbol{X}}}_{g}-\bar{\boldsymbol{X}} \right)\left( {\bar{\boldsymbol{X}}}_{g}-\bar{\boldsymbol{X}} \right)^{'}$$

where ${\bar{\boldsymbol{X}}}_{g}$ is the vector of covariate means for arm $g$ and $\bar{\boldsymbol{X}}$ the vector of overall means. The within-group sum of squares is:

$$\boldsymbol{E}=\sum_{g=1}^{4} \sum_{i=1}^{n_{g}} \left( \boldsymbol{X}_{gi}-{\bar{\boldsymbol{X}}}_{g} \right)\left( \boldsymbol{X}_{gi}-{\bar{\boldsymbol{X}}}_{g} \right)^{'}$$

Defining $\lambda$ as the vector of eigenvalues of $\boldsymbol{E}^{-1}\boldsymbol{B}$ and $r=\mathrm{rank}\left( \boldsymbol{B} \right)$ then Wilks’ Λ is $\prod_{i=1}^{r} \frac{1}{1+\lambda_{i}}$.
