# Supplementary material for: Evaluation of a policy intervention to promote the health and wellbeing of workers in small and medium sized enterprises – a cluster randomised controlled trial
Source: BMC Public Health. 2019 May 2;19:493. doi: 10.1186/s12889-019-6582-y (PMC6498586; doi:10.1186/s12889-019-6582-y)
Supplement: Supplementary file 5 — Estimated number of interviews per arm for process evaluation. (DOCX 12 kb) [file 12889_2019_6582_MOESM5_ESM.docx]

**Additional file 5**

| **Estimated number of interviews per intervention and control arm** | | | | | |
| --- | --- | --- | --- | --- | --- |
|  | **Intervention arm** | | | **Control arm** | |
|  | **‘Small’** | **‘Large’** | **Total** |  | **Total** |
| No of SMEs | 3 | 3 | 6 |  | 3 |
| No of interviews per SME | 3 | 6 | 9 |  | 4 |
| Total interviews | 9 | 18 | 27 |  | 12 |
|  |  |  |  |  |  |
| **Number of SMEs included in each measurement** | | |  |  |  |
|  | **I^1^** | **I^2^** | **C^1^** | **C^2^** | **Total** |
| Baseline measurement | 5 | 5 | 5 | - | 15 |
| Mid-term measurement | 5 | 5 | 5 | - | 15 |
| End-point measurement | 5 | 5 | 5 | 3 | 18 |
|  |  |  |  |  |  |
