# Supplementary material for: Evaluation of a policy intervention to promote the health and wellbeing of workers in small and medium sized enterprises – a cluster randomised controlled trial
Source: BMC Public Health. 2019 May 2;19:493. doi: 10.1186/s12889-019-6582-y (PMC6498586; doi:10.1186/s12889-019-6582-y)
Supplement: Supplementary file 6 — Specifications of statistical analysis planned for quantitative outcomes. (DOCX 13 kb) [file 12889_2019_6582_MOESM6_ESM.docx]

**Additional file 6**

We will collect data on individuals $i=1,\ldots,N$ nested in clusters (SMEs) $j=1,\ldots,J$ and trial arm $g=1,2,3,4$. The observations will be recorded at time $t=1,\ldots,T$. For a given dichotomous endpoint (the primary endpoint being the response to the question “Does your organisation…?” – see Section x.x), $y_{gijt}$, we also observe a three-level treatment indicator $\boldsymbol{D}_{gt}$ for control and two treatment conditions, a dichotomous ‘measurement’ indicator $m_{gt}$ (equal to one if the arm had been measured prior to time $t$ and zero otherwise), and a vector of dichotomous indicators for time period $T_{t}$. The model is specified as:

$$log(\Pr\left( y_{gijt}=1 | \boldsymbol{D}_{gt},m_{gt},\boldsymbol{T}_{t} \right))=\mu+\boldsymbol{D}_{gt}^{'}\boldsymbol{\delta}_{1}+\delta_{2}m_{gt}+\alpha_{j}+\theta_{jt}$$

$$\alpha_{j}\sim N\left( 0,\sigma_{\alpha}^{2} \right)$$

$$\theta_{jt}\sim N\left( \tau_{t},\sigma_{t}^{2} \right)$$

Where $\alpha_{j}$ is the between-cluster random effects term, $\theta_{jt}$ is the within cluster, between time period random effect, and $\tau_{t}$ are parameters to be estimated to capture any secular trends. To note, each SMEs is only observed for each of two time periods (pre- and post-intervention).Thus, the parameters to be estimated are $\Theta=[\mu,\boldsymbol{\delta}_{1},\delta_{2},\sigma_{\alpha}^{2},\sigma_{t}^{2} \tau_{1},\ldots,\tau_{T}]$. The treatment effects of primary interest are the two parameters $\boldsymbol{\delta}_{1}=\left[ \delta_{1,lo},\delta_{1,hi} \right]$ which correspond to the two levels of the incentive condition. These parameters can be interpreted as the log relative risk of the outcome compared to no incentive condition.

We will use “weakly informative” priors, which provide little information on the location of a parameter, but provide enough information to regularise the model and facilitate computation. In particular, $N\left( {0,10}^{2} \right)$ priors will be used for the parameters in the linear predictor and half-standard Cauchy priors will be used for hyper-parameter covariance terms.
